# Endoscopic Treatment of Obesity and Nutritional Aspects of Bariatric Endoscopy

**DOI:** 10.3390/nu13124268

**Published:** 2021-11-26

**Authors:** Jan Král, Evžen Machytka, Veronika Horká, Jana Selucká, Filip Doleček, Julius Špičák, Viktorie Kovářová, Martin Haluzík, Marek Bužga

**Affiliations:** 1 Department of Hepatogastroenterology, Institute for Clinical and Experimental Medicine, Vídeňská 1958/9, 14021 Prague, Czech Republic; evzen.machytka@ikem.cz (E.M.); jana.selucka@ikem.cz (J.S.); jusp@ikem.cz (J.Š.); 2 Faculty of Medicine and Dentistry, Palacký University Olomouc, 77147 Olomouc, Czech Republic; 3 Department of Internal Medicine-Gastroenterology and Geriatrics, University Hospital Olomouc, 77900 Olomouc, Czech Republic; 4 Research Obesity Centre, Department of Human Movement Studies, Faculty of Medicine, University of Ostrava, 70300 Ostrava, Czech Republic; veronika.horka@osu.cz; 5 Department of Surgical Studies, Faculty of Medicine, University of Ostrava, 70300 Ostrava, Czech Republic; filip.dolecek@gmail.com; 6 Department of Diabetology, Institute for Clinical and Experimental Medicine, Vídeňská 1958/9, 14021 Prague, Czech Republic; viktorie.kovarova@ikem.cz (V.K.); martin.haluzik@ikem.cz (M.H.); 7 Department of Physiology and Pathophysiology, Faculty of Medicine, University of Ostrava, 70103 Ostrava, Czech Republic; Marek.Buzga@osu.cz; 8 Institute of Laboratory Medicine, University Hospital Ostrava, 70800 Ostrava, Czech Republic

**Keywords:** obesity, nutrition, deficience, endoscopic bariatric and metabolic treatments (EBMTs), weight loss

## Abstract

Obesity is a significant problem worldwide. Several serious diseases that decrease patient quality of life and increase mortality (high blood pressure, dyslipidaemia, type 2 diabetes etc.) are associated with obesity. Obesity treatment is a multidisciplinary and complex process that requires maximum patient compliance. Change of lifestyle is fundamental in the treatment of obesity. While pharmacotherapeutic options are available, their efficacy is limited. Surgical treatment though highly effective, carries the risk of complications and is thus indicated mostly in advanced stages of obesity. Endoscopic treatments of obesity are less invasive than surgical options, and are associated with fewer complications and nutritional deficits. Currently, there is a large spectrum of endoscopic methods based on the principles of gastric volume reduction, size restriction and gastric or small bowel bypass being explored with only few available in routine practice. The aim of this publication is to present an up-to-date summary of available endoscopic methods for the treatment of obesity focusing on their efficacy, safety and nutritional aspects.

## 1. Introduction

Obesity is a serious disease that affects hundreds of millions of individuals worldwide. According to the WHO, obesity is defined as abnormal or excessive fat accumulation that presents a significant health risk, and is closely related to long-term complications and numerous associated diseases. Obesity in Caucasian populations is defined as a body mass index (BMI) higher than 30 kg/m^2^ or higher (Table 1) [1]. Obesity per se currently affects about one fourth to one third of people in the developed countries. The incidence of oobesity has nearly tripled since 1975. More than 1.9 billion patients were overweight and 650 million were obese in 2016. According to WHO statistics, more than 340 million children up to the age of 5 years are overweight or already obese [2]. Despite all the effort to fight obesity, its incidence is on the rise. The estimated annual cost of obesity and its complications is around 147 billion US dollars in the USA and 70 billion euros in Europe [3,4].

The pathophysiology of obesity is complex and involves genetic predispositions, environmental factors, and western lifestyle. The principal, interconnected factors include a sedentary lifestyle, processed food, high-calorie diet, insufficient physical activity, industrialization and economic growth. Obesity leads or significantly contributes to the development of a number of illnesses that impair quality of life and are associated with early mortality. They also considerably impact the health care system and economy. These diseases include diabetes mellitus (DM), arterial hypertension, liver steatosis, myocardial infarction, stroke, cancer, musculoskeletal disorders, psychiatric illnesses etc. [5,6,7,8].

The treatment of obesity is a long-term process that requires a multidisciplinary approach (nutrition counselling, endocrinology, gastroenterology, surgery, psychiatry, psychology, physiotherapy, and fitness coaching [9]. Patients´ compliance plays a fundamental role in such a process. Treatment is based on dietary measures specifically personalized to the given patient and regular physical activity that takes into account the patient´s weight, age, and fitness. Other treatment options include pharmacotherapy (orlistat, naltrexone-bupropion, liraglutide and others), which is of limited efficacy and with which only a small number of patients manage to reduce their weight by at least 10% [10]. Pharmacotherapy may influence fat absorption (orslistat), dopamine and noradrenaline re-uptake in the central nervous system (bupropion), and block opioid receptors (naltrexone) thus acting on the POMC loop, or it may increase satiety and decrease hunger through stimulation of glucagon-like peptide-1 receptors (liraglutide) [11]. Surgical treatment (gastric binding, sleeve gastrectomy, bypass surgery, Roux-en-Y gastric bypass, biliodigestive anastomosis etc.) is the most effective option of obesity treatment with an average total weight loss of 15–35%. However, bariatric surgery is less accessible, carries the risk of complications and represents a financial burden [12,13]. According to current recommendation, bariatric surgery is indicated in Class III obese patients and in Class II obese patients with comorbidities (type 2 DM, etc.). 

Endoscopic Bariatric and Metabolic Therapies (EBMT) represent another progressive alternative that offers higher efficacy than pharmacotherapy and at the same time is less invasive and has a lower incidence of complications than classical surgical treatment. In some procedures, the cost of endoscopic treatments may be lower than those of bariatric surgery. Efficacy in terms of weight loss achieved is around 10–25%. Previous studies have demonstrated that long-term total body weight reduction by 5–10% is sufficient to achieve a significant decrease in the risk of a cardiovascular event or type 2 DM [14,15].

As in the case of bariatric surgery, bariatric endoscopy is experiencing relatively rapid developments. Endoscopic methods were considered a marginal treatment option for a long time. This view has changed significantly over the past five years. An important step forward was the inclusion of these therapeutic methods in the guidelines of the American Association of Clinical Endocrinologists (AACE), The Obesity Society (TOS), and the American Society for Metabolic and Bariatric Surgery (ASMBS) in 2019 [16]. 

The method of Endoscopic Bariatric and Metabolic Therapies (EBMT) predominantly involve the stomach, but they also include procedures that remodel the duodenum or small bowel. Systematic classification of procedures as those used in the IFSO guidelines is not as yet unified in bariatric surgery [17]. An overview of three American specialty societies published in 2019 represents the first official classification of these methods from the aspect of specialty organizations. The recommended methods are gastric volume reduction including intragastric balloons (IGB), gastric endoscopic remodeling using plication, and thirdly, reduction of calorie intake using aspiration therapy. This classification is based on the methods registered in the USA by the FDA. On the other hand, there are numerous methods today that lack American FDA registration but are registered and approved within the European Union, or are approved in the context of experimental studies [18,19].

As in the case of surgical methods, endoscopic methods today are perceived not only as restrictive procedures but also as procedures with an important metabolic effect [20]. In this context, it is important which section of the GIT is involved in the given procedure and how this procedure alters the physiological conditions or function of the given section. Seen from this perspective, a whole range of procedures involving the stomach ranging from balloon implantation to endoscopic plication is currently used or being developed. Procedures involving the duodenum and small bowel aimed at producing a metabolic effect in the treatment of type 2 DM must also be considered [21,22]. 

### Endoscopy Bariatric Procedures

Based on recent publications and guidelines, endoscopy bariatric procedures are divided into four parts (1) procedures that reduce gastric volume (2) procedures that slow gastric emptying (3) bypass procedures, and (4) other procedures.

## 2. Procedures That Reduce Gastric Volume

### 2.1. Intragastric Balloons (IGB)

**Figure 1:**
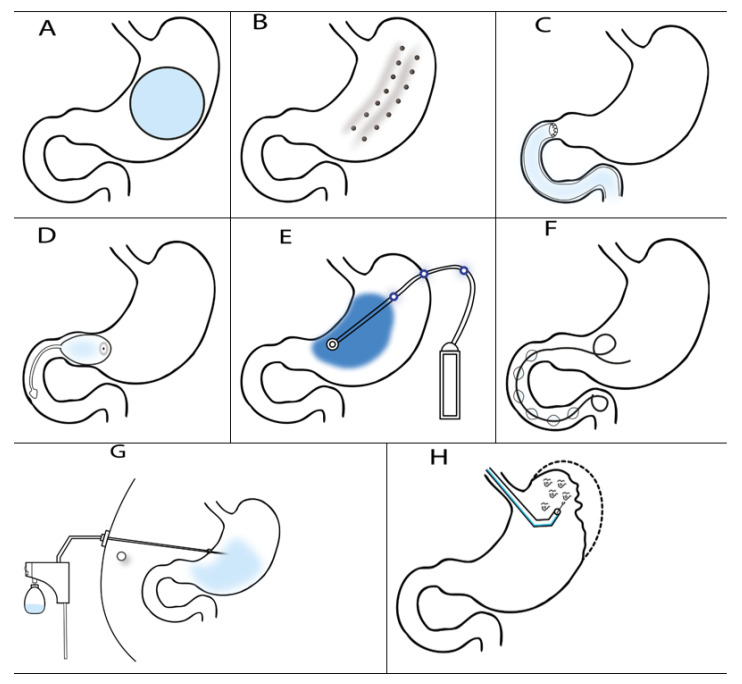
Overview of endoscopic bariatric procedures: (**A**) intragastric balloon placement, (**B**) endoscopic sleeve gastroplasty, (**C**) duodenojejunal bypass, (**D**) TransPyloris Shuttle, (**E**) electrical stimulation, (**F**) SatiSphere, (**G**) aspiration therapy, (**H**) Primary Obesity Surgery Endoluminal (POSE).

IGB (Figure 1A) is a procedure for a gastric volume reduction. The mechanism of action involves stimulation of mechanoreceptors in the stomach, which then stimulates the vagus nerve sending signals to the hypothalamus that subsequently induces the feeling of fullness and at the same time slows gastric emptying. The first IGB was implanted in 1982 by Nieben and Harboe in Denmark [23]. The first intragastric balloon in the United States was introduced in 1985—Garren-Edwards gastric bubble [24]. Since then, a whole range of IGB of various sizes and shapes has been introduced into standard clinical practice (Table 2). Most IGBs are oval or round and have a volume of 400–700 mL. IGBs may be filled with a fluid, a gas or a combination of both [25,26,27,28]. The Reshape Duo™ (ReShape Lifesciences™, San Clemente, CA, USA) uses two balloons in order to decrease the risk of balloon migration [29]. IGBs are manufactured by a number of companies and are most often placed under endoscopic control while the patient is sedated, or they may be swallowed in the form of a capsule. They are usually placed for a period of 6 months. After this, the IGB is subsequently removed endoscopically, or is spontaneously excreted through the GIT. IGBs in the form of a capsule also exist (e.g., Elipse^®^, Allurion, Natick, MA, USA; Obalon^®^, Obalon Therapeutics, Carlsbad, CA, USA; Ullorex^®^, Obalon Therapeutics). In the case of the Elipse^®^ balloon, the suture material closing the balloon´s valve is degraded after a set period of time (1–4 months) and the balloon is evacuated and leaves the body spontaneously. The Obalon^®^ balloon is extracted endoscopically [20,30]. The volume of the Spatz™ balloon (Spatz FGIA, Great Neck, NY, USA) may be adjusted (increased, decreased) during its implantation via an extractable catheter. Reduction of the IGB volume at the beginning of treatment reduces the number of early IGB extractions due to intolerance. In contrast, greater IGB volume increases treatment efficacy. 

Tate et al. [31] performed an analysis of 8 randomised trials that compared total body weight loss (% total body weight loss, TBWL) between patients with an implanted IGB and a control group. In five studies, where the IGB was implanted for 6 months, the average %TBWL was 9.7% compared to 5.6% in the control groups. However, the average incidence of serious complications was high (10.5%) (permanent vomiting, stomach-ache, GERD, ulcers, or perforation) [31]. The Vargas et al. study evaluated the safety, efficacy, and tolerance of the IGB (Orbera^®^) in a total of 321 patients. In the sixth month, %TBWL of 5%, 10% and 15% was achieved in 88%, 62% and 31% of patients, respectively. The results also showed a decrease in cholesterol, triglyceride, and glycated haemoglobin (HbA1c) levels as well as improved compensation of arterial hypertension. This trial confirmed the efficacy and safety of IGBs and no serious complications following IGB placement were reported [32]. A retrospective study comparing efficacy and safety between IGB and endoscopic sleeve gastroplasty (ESG) was published in 2019. The study included a total of 47 patients with IGB and 58 patients with ESG. In the IGB group, the initial BMI was lower (34.5 kg/m^2^) than in the ESG group (41.5 kg/m^2^). In the IGB group, the average TBWL in the first, third and sixth month was significantly lower than in the ESG group (6.6% vs. 9.9%; *p* < 0.001; 11.1% vs. 14.3%; *p* < 0.004; 15% vs. 19.5%; *p* < 0.01). The IGB group also recorded a higher number of adverse events than the ESG group did (17% vs. 5.2%; *p* < 0.048). Both methods were effective in reducing body weight, but ESG was effective with a lower number of complications [33]. One significant drawback of IGB is the risk of recurrent weight gain once the balloon is extracted. This fact was shown by the Herve et al. study, where in 100 patients the average %EWL following extraction was 36.8% and the %EWL was only 26.8% 12 months after explantation [34,35].

### 2.2. TransPyloric Shuttle (TPS)

The TransPyloric Shuttle (TPS; BAROnova, Goleta, USA) (Figure 1D) consists of a silicone balloon that is anchored in the region of the pylorus. This balloon is attached to a silicone catheter that is inserted into the duodenum and has a smaller oval balloon at its tip. The advantages include more rapid filling of the balloon and a slower gastric emptying. Marinos et al. published a trial in 2014 that included 20 patients with TPS. The average BMI of these patients was 36.0 kg/m^2^. A TPS was implanted for 3 or 6 months. In patients with a TPS implanted for 3 months, the excess weight loss in percentage (%EWL) was 25.1% in total, and the average total body weight loss (%TBWL) was 8.9%. In patients with a TPS implanted for 6 months, the %EWL was 41% and the %TBWL was 14.5% [36]. The ENDObesity^®^ II trial was started in 2015—TransPyloric Shuttle^®^ System for Weight Loss (ClinicalTrials.gov-NCT02518685). A total of 270 patients were enrolled in the study, but the results have not yet been published. The TPS has been approved for clinical use by the US regulatory agencies since 2019. 

### 2.3. SatiSphere

SatiSphere (EndoSphere Inc., Columbus, OH, USA) (Figure 1F) is a device consisting of a nitinol guidewire with mounted polyethylene terephthalate oval balloons. The device is implanted into the pylorus and duodenum endoscopically under general anesthesia. The principle lies in the decrease of food intake associated with delayed transit time of nutrients and food through the duodenum. In 2013, Sauer et al. published a study that included a total of 31 patients (21 patients underwent implantation, 10 patients represented the control group). SatiSphere was implanted for a total period of 3 months, after which the maximum weight reduction was 6.7 kg and the %EWL was 18.4%. Migration of the device occurred in ten patients out of 21 and two patients required emergency surgery. This led to termination of the study [37].

## 3. Restrictive Procedures

### 3.1. Endoscopic Sleeve Gastroplasty (ESG)

ESG is an endoscopic method (Figure 1B) that aims to imitate surgical sleeve gastrectomy thus leading to gastric volume reduction and induction of early fullness. This procedure is performed under general anesthesia using an video-endoscope and a corresponding device (Endo Tools Therapeutics Endomina, Charleroi, Belgium; Apollo Endosurgery OverStich™ Endoscopic Suturing System, Winter Park, FL, USA) that places sutures through all the layers of the stomach proximally from the angular notch. A prospective multicenter trial conducted by Barrichello et al. involved a total of 193 patients from 7 centres who underwent ESG (Apollo OverStich™, Jeddah, Saudi Arabia). In these patients, the average %TBWL 6 months after the procedure was 14.25% ± 5.26% and the %EWL was 56.15% ± 22.93% (*p* < 0.05). After one-year follow-up, the %TBWL was 15.06% ± 5.22% and the %EWL was 59.41% ± 25.69% (*p* < 0.05). Serious complications were recorded in 1% of patients [38]. Lopez-Nava et al. published the results of a study dealing with the efficacy and safety of ESG (Apollo OverStich™). This was a prospective monocentric study of 154 patients. After two years of follow-up, the average BMI fell from 38.2 to 30.8 kg/m^2^, the %TBWL was 19.5% and the %EWL was 60.4%. No adverse event was reported in this group [39].

Huberty et al. published a randomized trial that enrolled a total of 71 patients (49 patients, 22 controls) who underwent endoscopic gastroplication (Endomina, Endo Tools Therapeutics, Belgium). Comparison of both groups has shown that the %EWL value 6 months from the procedure was significantly higher in the gastroplication group than in the control group (38.6% vs. 13.4%; *p* < 0.001). In addition, the quality of life was significantly better in the Endomina group than in the control group (52.8% vs. 42.1%). No serious adverse events were recorded during the trial [40]. 

### 3.2. Primary Obesity Surgery Endoluminal (POSE)

POSE is a bariatric method that uses a flexible endoscope and a minimally invasive surgical approach (per-oral Incisionless Operating Platform™, USGI Medical, San Clemente, CA, USA). The aim of POSE is to decrease gastric volume and thus induce a more rapid feeling of fullness. The procedure is performed under general anesthesia. It involves endoscopic plication of the stomach in the region of the fundus and distal body. In 2015 Lopez-Nava et al. published a study evaluating the efficacy and safety of the POSE system. This study included a total of 147 patients who were followed-up for a period of one year. The initial BMI was on average 38.0 ± 4.8 kg/m^2^. A total of 116 patients completed the study. One year after the procedure, the %TBWL was 15.1% ± 7.8% and the %EWL was 44.9% ± 24.4%. No adverse events were reported in this study [41]. Espinós et al. also published their experience with the POSE system. Their study enrolled a total of 45 patients. BMI at the beginning of the trial was 36.7 ± 3.8 kg/m^2^ on average. After 6 months of the follow-up, the average BMI was 31.3 ± 3.3 kg/m^2^, the %TBWL was 15.5% and the %EWL was 49.4%. The average duration of the procedure was 69.2 ± 26.6 min [41]. Sullivan et al. randomized a total of 332 patients (POSE = 221, sham = 111). The results of this study were notably inferior; the %TBWL 12 months from the procedure was 4.95 ± 7.04% in the POSE group compared to 1.38 ± 5.58% in the control group. Serious complications associated with the procedure itself were recorded in 5% [42]. A definitive assessment of this procedure will depend on the results of further studies [43].

### 3.3. Transoral Gastroplasty

Transoral gastroplasty using the TOGA system (Satiety Inc., Palo Alto, CA, USA) consists of capturing the mucosa in the region of the stomach´s lesser curve using high-powered suction, placement of a vertical suture (approx. 8 cm) and subsequent creation of a small pouch in the proximal section of the stomach [44]. In 2008 Deviere et al. published the first study that evaluated the efficacy and safety of the TOGA system. It included a total of 21 patients. The procedure was uncomplicated in all patients. One month later, the average %EWL was 16.2%, and after 6 months the average %EWL was 24.4%. The most frequent adverse events reported included vomiting, pain, nausea, and transient dysphagia [45]. Another study dealing with the efficacy and safety of the TOGA system was published by Moreno et al. This group enrolled a total of 11 patients. The procedure was uncomplicated in all of them. The average %EWL one month after the procedure was 19.2% and in the sixth month the average %EWL was 46.0%. The average decrease in BMI was from 41.6 kg/m^2^ before treatment to 33.1 kg/m^2^ six months later [46].

## 4. Bypass Techniques

### 4.1. Duodenojejunal Bypass (DJB)

DJB is an endoscopic technique (Figure 1C) that involves the insertion of a 60 cm Teflon EndoBarrier^®^ (GI Dynamics, Lexington, KY, USA) sleeve and its anchoring in the bulb. The mechanism of action involves imitation of a gastric bypass (food from the gastric pouch passes through the impenetrable sleeve directly into the jejunum without being in contact with duodenal mucosa), thus eliminating the duodenum functionally but without altering the anatomical relationships as in the case of a surgical gastric bypass. A multicenter randomized study was published in 2014 evaluating the efficacy of the EndoBarrier^®^ (EB) system in the treatment of type 2 DM and obesity compared to dietary measures. A total of 34 patients underwent implantation of EB and 39 patients were allocated to the control group. After six months, the group of patients with EB recorded a significantly greater decrease in %EWL (32.0% vs. 16.4%, *p* = 0.05). There was also an improvement in glycated hemoglobin levels in these patients (7.0% vs. 7.9%, *p* = 0.05) [47]. In another study, Forner et al. enrolled a total of 114 patients. The average follow-up was 51 weeks. The average %TBWL was 10.5%. An adverse event was recorded in a total of 74% of patients, including serious complications (6x system obstruction, 5× bleeding, 2× liver abscess, and 1× acute pancreatitis). The weight loss following EB implantation was variable and the frequency of adverse events meant that the use of this device in clinical practice was terminated [48]. In our study with EB, we included thirty obese patients with poorly controlled T2DM who underwent the EB implantation and were assessed before and 1, 6 and 10 months after the implantation, and 3 months after the removal of DJBL. The implantation decreased body weight by 12 kg, and improved lipid levels and glucose regulation along with reduced glycemic variability. White blood cell counts slightly increased and red blood cell counts decreased throughout the EB implantation period along with decreased ferritin, iron and vitamin B12 concentrations. Blood count returned to baseline values 3 months after EB removal. Decreased body weight and improved glucose control persisted with only slight deterioration 3 months after EB removal while the effect on lipids was lost [49].

### 4.2. Gastroduodenojejunal Bypass

This type of procedure involves insertion of a sleeve (ValenTX Inc., Maple Grove, MN, USA) into the bowel lumen. This sleeve is fixed in the region of the gastroesophageal junction, and this leads to food bypassing the stomach, duodenum and jejunum [30]. Only one study assessing the device´s efficacy and safety has been published to date. This was a prospective monocentric study, where the device was implanted in a total 13 patients for a period of one year. The average BMI of these patients was 42 kg/m^2^. One year from implantation, the %EWL was on average 54%. This study showed that the device is well tolerated, and no complications related to its implantation were reported [50].

## 5. Aspiration Therapy

Aspiration therapy (Aspire Assist^®^, Aspire Bariatrics, King of Prussia, PA, USA) is based on the implantation of a percutaneous endoscopic gastrostoma which the patient uses to aspirate approximately 30% of the stomach content 20 min after a meal. The principle involves aspiration of part of the food ingested, thus reducing and controlling the amount of calories absorbed. A pilot study from the USA conducted by Sullivan et al. (2013) attempted to evaluate the efficacy of aspiration therapy compared to lifestyle and diet adjustments. A total of 18 patients were randomized in this study (2:1). The Aspire Assist^®^ was inserted in 11 patients. Four patients out of 7 from the second group completed the one-year follow-up. One year after aspiration therapy, there was a decrease in %EWL of 49% ± 7.7% compared to the second group where the %EWL decreased by 5.9% ± 5.0%, (*p* = 0.021). Noren et al. published a study in 2016 that involved 25 patients. The average BMI before therapy was 39.8 kg/m^2^. One year after treatment, the average BMI was 32 kg/m^2^ and the %EWL was 54.4%. No complications were reported. Treatment compliance during the first year was 80% [51]. Thompson et al. published a study in 2019 that summarized their 4-year experience with aspiration therapy. This was a multicenter randomized trial conducted in 10 centers in the USA. A total 171 patients were randomized into two groups (aspiration therapy and lifestyle adjustments vs. lifestyle adjustments). Eighty-two patients completed the first year of treatment in the aspiration therapy group and 58 completed 4 years of treatment. The average BMI of patients before the study started was 41.6 ± 4.5 kg/m^2^. At the end of the first year, the average BMI in the aspiration group was 34.1 ± 5.4 kg/m^2^ and the decrease in %TWL was 18.3 ± 8.0%. After 4 years of the trial, the %TWL was 18.7%. Only two complications were recorded during the whole duration, and both were resolved by extracting the device. This study demonstrated that aspiration therapy is a safe and effective treatment for patients with class II and III obesity [52].

## 6. Gastric Electrical Stimulation (GES)

Retrograde electrical stimulation (Figure 1E) is an innovative stimulation technique that uses two electrodes placed in the proximal section of the pylorus. Stimulation leads to a decrease in water and food intake and slows gastric emptying. The study performed by Zhang et al. evaluated the effect of electrical stimulation on food intake, gastric accommodation and emptying in obese patients. A total of 16 patients with an average BMI of 32.1 kg/m^2^ were enrolled in the study. The median gastric emptying was 113 min compared to the sham group (106 min). The average amount of food that led to fullness was 490 mL in the stimulation group and 580 mL in the sham group. This study showed that stimulation leads to decreased gastric accommodation and a reduction in calorie intake [53]. Another gastric stimulation method is the Tantalus system (MetaCure Ltd., Orangeburg, NY, USA). Bohdjalian et al. published a study evaluating its effect on obese patients and type 2 DM. A total of 13 obese patients with type 2 DM were enrolled in the study. Three months after implantation, there was a decrease in HbA1c from 8.0 ± 0.2% to 6.9 ± 0.1% (*p* = 0.05) and a reduction in weight from an initial 104.4 ± 4.4 kg to 99.7 ± 4.8 kg [54] (*p* = 0.05). A systematic review by Rayna Cha et al. (2014) [55] evaluated a total of 31 studies involving gastric electric stimulation. These showed that weight loss occurred during the first 12 months but only a minority of these studies had a follow-up of more than one year. GES thus appears to be a promising innovative method in the treatment of obesity. However there are no long-term efficacy results as yet [55].

## 7. Other Methods

### 7.1. Duodenal Mucosa Resurfacing (DMR) 

Duodenal mucosa resurfacing is a technique primarily designed for the treatment of type 2 DM and non-alcoholic fatty liver disease (NAFLD). Studies have shown that increased fat and carbohydrate intake leads to duodenal mucosa hypertrophy and a high concentration of enteroendocrine cells associated with high secretion of GIP that consequently leads to insulin hypersecretion and insulin resistance [56]. DMR involves circular hydrothermal ablation of the duodenal mucosa and its subsequent regeneration, following which the endocrine cells renew their function and incretin secretion. The first DMR trial was published in 2016 by Rajagopalan et al. (proof-of-concept). Twenty-eight patients underwent long ablation (9.3 mm on average), and 11 patients underwent short ablation (3.4 cm on average). The procedure was well tolerated—two patients developed stenosis of the duodenum that was subsequently successfully dilated. In these patients, there was a decrease in HbA1c on average of 1.2% over a period of 6 months [57]. A recent prospective multicenter study conducted by Baar et al. evaluated the effect of DMR (Fractyl Laboratories) on the treatment of type 2 DM. This study included a total of 46 patients, 37 of which completed the procedure. An adverse event occurred in 52% of patients, mainly involving minor complications and only one was serious. Six months after the procedure, HbA1c decreased on average by 0.9% ± 0.2% and weight decreased on average by 2.5 ± 0.6 kg. The results after twelve months were comparable to those after six months. This study showed that DMR is an endoscopic technique that leads 7.2 to improved diabetes control in patients with type 2 DM treated with oral hypoglycaemic agents [58]. However, possible drawbacks of this method must be kept in mind, such as duodenal stenosis that developed in three patients and was successfully resolved by endoscopic balloon dilation. It appears that the efficacy of this method depends on the length of the ablation, which may vary between patients for anatomical reasons. 

### 7.2. The Incisionless Magnetic Anastomotic System (IMAS)

This technique involves the creation of an anastomosis using two simultaneously endoscopically delivered magnets. In bariatric procedures, one magnet is placed in the jejunum and the other in the ileum [59,60]. Machytka et al. performed a pilot study that enrolled a total of 10 patients with an average BMI of 41 kg/m^2^ who all underwent magnetic anastomosis. The average %TBWL was 14.6% and there was also a decrease in glycated hemoglobin levels in all diabetics by 1.9% [61]. However, this is a technically exacting method and laparoscopic assistance was necessary in all cases to create the anastomosis. 

### 7.3. Botulinum Toxin A 

This procedure involves an application of botulinum toxin A via an injector into the area of the gastric antrum and/or fundus. This then delays gastric emptying and induces early fullness. The effect of this treatment is limited to a period of 3–6 months. A meta-analysis by Bang et al. (2015) [62] evaluated the results of 8 studies that included a total of 115 patients (79 application of botulinum toxin, 36 placebo). The weight of patients who underwent botulinum toxin A application decreased compared to that of the placebo group. However this meta-analysis did not report either %TBWL or %EWL [62].

## 8. Nutrition

### 8.1. Nutrition and Dietary Recommendations for Bariatric Endoscopy

Adverse effects of bariatric surgery are not uncommon; thus, efforts should be made to reduce their incidence. Many studies have focused on the surgical, digestive, and nutritional complications of bariatric surgery [63,64,65]. On the other hand, very few studies have assessed nutritional deficiencies following non-surgery (endoscopic) bariatric procedures.

Nutritional deficiencies have been described after malabsorptive surgery. However, deficiencies from restrictive surgery have only recently been reported [66]. Although several cases of acute nutritional complications have been reported after bariatric surgery, monitoring of the nutritional deficiencies responsible for these complications is not routinely performed in clinical practice [65]. In a systematic review, Kaidar-Person showed that the prevalence of nutrient deficiencies in obese individuals is higher than in healthy individuals [67,68]. The literature suggests that bariatric surgery patients are at risk for being deficient in the following nutrients after surgery: vitamins B12, B1, C, folate, A, D, and K, along with the trace minerals iron, selenium, zinc, and copper [69].

### 8.2. Specifics of Nutrition in Bariatric Endoscopic Surgery

The type of bariatric surgery changes the nature of the diet, and there is a postoperative recommendation with standardized loosening that was published in 2019 [16]. Recommendations for bariatric endoscopy can then be derived from these guidelines which are not yet available. It can be expected that they will be similar, in some cases not as strict as those after surgery. Standard recommendations are that a liquid diet should be introduced within 24 h of surgery, with a gradual transition to a slurry and a solid diet according to individual tolerance. Initially, patients are advised to take 3 small meals a day that are thoroughly chewed, then, according to tolerance, it is recommended that they switch to a reduction diet according to the principles of healthy eating [70].

### 8.3. Nutritional Deficiency

Among bariatric surgery patients, the most frequently reported vitamin deficiencies that make them vulnerable to the development of anemia are deficiencies of vitamin B12 and folate. Vitamin B12 uptake after restrictive procedures such us sleeve gastrectomy can become inadequate due to a lower production of hydrochloric acid, which is needed to release bound vitamin B12 in food [71,72]. Endoluminal methods such as endoscopic sleeve gastroplasty or intragastric balloons are also restrictive methods, but they do not reduce the hydrochloric acid-producing part of the stomach and intrinsic factors. The type of diet consumed or a previous vitamin B12 deficiency will then be an important factor.

Vitamin B1 deficiency is a concern in bariatric patients because in the most severe cases it can be responsible for neurological complications such us Wernicke’s encephalopathy and periferial neuropathy [73]. These complications have been observed after restrictive and malabsorbtion procedures [74]. Severe vitamin B1 deficiency is a dangerous complication but its prevalence is relatively low [75]. Vitamin B6 plays a role in amino acid metabolism, gluconeogenesis, and neurotransmitter synthesis; thus, it is important to ensure adequate levels [76]. Evidence of its deficits is inconsistent. In a study using endoscopic sleeve gastroplasty no patient was deficient in this vitamin. Similar data have been presented in studies of patients after LSG and Roux-en-Y gastric bypass [72]. By contrast, Damms-Machado et al. reported vitamin B6 deficiencies after LSG, which has not been shown by others [77]. 

Vitamin D or cholecalciferol is important for weight maintenance and for bone metabolism [78]. Patients who qualify for obesity surgery present with vitamin D insufficiency, with many being deficient and others having secondary hyperparathyroidism. Vitamin D status may worsen after obesity surgery, even when supplemental calcium and vitamin D are prescribed [79]. The cause of vitamin D deficiency in obesity is not well understood. It has been proposed that a low vitamin D status might be due to increased vitamin clearance from serum and an enhanced storage of vitamin D in adipose tissue [80]. Synthetic supplementation is a routine in both preoperative and postoperative periods, averaging 3000 IU per day [81]. 

It is indisputable that good nutritional status supports and improves the patient′s condition after bariatric surgery. Future prospective studies in different types of bariatric endoscopic procedures are needed.

## 9. Conclusions

Obesity and its associated complications represent a worldwide problem. Current western lifestyles with minimal physical activity and high calorie intake continue to increase the incidence of this disease and proportionately the costs associated with its treatment and that of its associated complications [82]. 

The treatment of obesity is a multidisciplinary process involving a whole range of specialists. Such treatment must be tailored, and patients must be motivated as their long-term cooperation is vital. Four principal modalities may be used in the treatment of obesity: lifestyle adjustments (energy intake and output, potential psychological or psychiatric intervention), pharmacological, endoscopic, and surgical treatments. 

Endoscopic treatment of obesity is a rapidly developing field in digestive endoscopy. We have a whole spectrum of endoscopic procedures at our disposal, ranging from the simple outpatient placement of an intragastric balloon to more complex procedures such as gastroplication or magnetic anastomosis. Several new devices are being developed and are currently being tested in clinical trials in order to assess their safety and efficacy before their approval for clinical use by the relevant regulatory bodies.

Endoscopic treatment is minimally and in most cases, procedures can be performed in an outpatient setting with the use of sedation or only short general anesthesia. This treatment does not leave any scars on the abdomen and does not induce the development of adhesions within the abdominal cavity. A major advantage in many cases is the reversibility of these procedures and surgical treatment may follow in all such cases if necessary (this is difficult if not impossible the other way around). The incidence of complications is low and serious complications are an exception including the rare occurrence of significant nutritional deficiencies. However, endoscopy is less effective compared to classical surgery, but this may not be an obstacle in certain patients. Lower efficacy may represent an advantage in situations where it is sufficient, such as in less obese patients with complications. The first results have demonstrated a positive effect of endoscopic treatment in cases of diabetes complications and non-alcoholic fatty liver disease. Long-term efficacy may be limited, typically in the case of temporary methods. Large, robust multicenter randomized studies will show the true and long-term efficacy of these new methods as well as their potential drawbacks and deficiencies. Nonetheless, endoscopic treatment is an integral part of the complex treatment of obesity today.

## Figures and Tables

**Table 1:** BMI chart.

Classification	BMI (kg/m^2^)
Underweight	<18.5
Normal weight	18.5–24.9
Overweight	25.0–29.9
Obese class I	30.0–34.9
Obese class II	35.0–39.9
Obese class III	≥40

**Table 2:** Types of intragastric balloons.

Type of Balloon	Volume	Filling	Material	Duration of Implantation	Form of Implantation
Orbera™	400–700 mL	normal saline with methylene blue		6 months	Gastroscopic
Heliosphere	550 mL	air		6 months	Gastroscopic
Medsil	400–700 mL	normal saline with methylene blue		6 months	Gastroscopic
ReShapeDuo™	900 mL	normal saline with methylene blue		6 months	Gastroscopic
Silimed	250–700 mL	normal saline with methylene blue		6 months	Gastroscopic
Spatz™	700 mL max.	normal saline with methylene blue		12 months	Gastroscopic
Elipse^®^	550 mL	normal saline with methylene blue		3 months	swallowed balloon
Obalon^®^	250–450 mL	Air		4 months	swallowed balloon
Ullorex^®^	300 mL	carbon dioxide		1 month	swallowed balloon
